# Supplementary material for: Making sense of unfamiliar COVID-19 vaccines: How national origin affects vaccination willingness
Source: PLoS One. 2021 Dec 29;16(12):e0261273. doi: 10.1371/journal.pone.0261273 (PMC8716032; doi:10.1371/journal.pone.0261273)
Supplement: Attachment — Submitted filename: Response to Reviewers.docx [file pone.0261273.s001.docx]

**Axel Pfleger**

Scientific Staff

Sigmund Freud PrivatUniversität Berlin
Columbiadamm 10, Turm 9
12101 Berlin

Email:	[axel.pfleger@sfu-berlin.de](mailto:axel.pfleger@sfu-berlin.de)

Tel.:	+491731635404

2021-11-12

Dear Dr Tsuzuki, dear reviewers,

Many thanks for your feedback on our manuscript. This rebuttal letter is to address each of your points. We have made amendments to the paper, but we have also commented on some of the points made in the review.

Regarding the editor’s comments, we have added a statement about all research funding sources to the manuscript, including the indicated statement about additional external funding. The cover letter was amended to include the funded statement as well.

**Addressing the 1st comment by reviewer #1:**

We have added clarifications wherever it was appropriate that nationalism/in-group biases may be one of many factors influencing vaccine decision-making. However, the comment about other factors such as vaccine technology and efficacy does not apply as we did specifically control for the vaccines’ technology when testing hypothesis 1. Additionally, when testing hypothesis 3, we isolated the factor ‘vaccine origin’ by testing for differences in vaccination willingness within each vaccine (control group vs. treatment group), not across vaccines. Dror and colleagues [8] have also come to similar conclusions, as noted in the introduction, where we have added a small point for clarification of this. We additionally added clarifying prose in the discussion related to the aspect of similar technologies and efficacies. The last point we want to highlight regarding this comment is that in the discussion, we already explicitly stated that, “[v]accine decision-making happens within a complex system of interconnected components, such as the underpinning vaccine technology, vaccine delivery, and one’s own background assumptions and viewpoints which is composed of various aspects (e.g., education, disease epidemiology, location within the social structure) […]. Trust, in particular, functions as a mediator within vaccine decision-making […].”

**Addressing the 2nd comment by reviewer #1:**

In-group bias can very much arise from perceptions of individual countries’ socio-political circumstances. As an important example of in-group biases, we actively included different countries’ favourability ratings, and additionally tested the factor ‘nationalism’ among countries with similar socio-political circumstances as well as similar vaccine technologies and efficacies (Germany vs. France).

**Addressing the 3rd comment by reviewer #1:**

We investigated in-group preferences for COVID-19 vaccination in Germany and found that indeed, vaccine willingness is partially dependent on in-group biases. The results show that in Germany, vaccine willingness is skewed towards German vaccines and vaccines from socio-political allies. This of course does not mean that people in other countries will have the same in-group biases as in Germany. In our manuscript, we clearly refer to the observed biases as respective to Germany, e.g.: “This study is designed to further explore the role of national origin from the perspective of citizens in Germany.” We did, however, replace the last two sentences in the discussion to highlight this issue. On the topic of other factors influencing vaccine decision-making, please see our response to the first comment about controlling for such factors and isolating the factor ‘national origin’.

**Addressing the 1st comment by reviewer #2:**

We believe this comment has also been addressed by our reply to the first reviewer’s first comment.

**Addressing the 2nd comment by reviewer #2:**

We added the following prose before the referenced section in the comment: “It should be critically noted, however, that people in Germany are in the privileged position of being able to choose between different vaccines. That a national vaccine is among the choice options is also not a given. In countries where vaccines are scarce and difficult to access, and where national products are not available, the role of nationalism is certainly of a different nature. Further studies are needed to clarify which factors significantly influence vaccination decisions here.”

**Addressing the 3rd comment by reviewer #2:**

This appears to be a display error on the reviewer's computer or something similar. Otherwise, we do not know what the reviewer is referring to in this comment. There are no abbreviations in the table titles.

Thank you very much for your time.

Sincerely,

**Axel Pfleger, Eric Jensen, Brady Wagoner, Lisa Herbig, and Meike Watzlawik**
**Sigmund Freud PrivatUniversität Berlin**
